# The Genetic Basis of Phenotypic Heterogeneity in the Neuronal Ceroid Lipofuscinoses

**DOI:** 10.3389/fneur.2021.754045

**Published:** 2021-10-18

**Authors:** Emily Gardner, Sara E. Mole

**Affiliations:** MRC Laboratory for Molecular Cell Biology and Great Ormond Street Institute of Child Health, University College London, London, United Kingdom

**Keywords:** neuronal ceroid lipofuscinosis, batten disease, NCL, CLN, mutation, gene, lysosomal disease

## Abstract

The neuronal ceroid lipofuscinoses (NCLs) are a group of inherited neurodegenerative disorders that affect children and adults. They share some similar clinical features and the accumulation of autofluorescent storage material. Since the discovery of the first causative genes, more than 530 mutations have been identified across 13 genes in cases diagnosed with NCL. These genes encode a variety of proteins whose functions have not been fully defined; most are lysosomal enzymes, or transmembrane proteins of the lysosome or other organelles. Many mutations in these genes are associated with a typical NCL disease phenotype. However, increasing numbers of variant disease phenotypes are being described, affecting age of onset, severity or progression, and including some distinct clinical phenotypes. This data is collated by the NCL Mutation Database which allows analysis from many perspectives. This article will summarise and interpret current knowledge and understanding of their genetic basis and phenotypic heterogeneity.

## Introduction

The neuronal ceroid lipofuscinoses (NCL), also known as Batten disease, are a group of inherited neurodegenerative life-limiting diseases that share some common clinical features including epileptic seizures, progressive psychomotor decline, and visual failure, and the accumulation of autofluorescent storage material. NCL usually begins in childhood, and most are inherited in an autosomal recessive manner. More than a dozen genes have been linked to families diagnosed with NCL (Table 1) (1). It is likely that most genes causing NCL have been identified.

**Table 1:** Summary of genotype and phenotype in NCL.

**Gene**	**Disease name(s)**	**Protein and location**	**Number of reported mutations**	**Widespread common mutations**	**Regional-specific mutations**	**Genotype-phenotype correlation**	**Age of onset**	**Presenting and typical clinical features**
*CLN1* *PPT1*	CLN1 disease	Palmitoyl protein thioesterase 1; lysosome	71	c.364A > T/p.(Arg122Trp); c.451C > T/p.(Arg151*)	c.223A > C/p.(Thr75Pro); c.29T > A/p.(Leu10*) in Scotland	**Infantile** Late infantile Juvenile Adult	**6–18 mo** 2–4 y 5–10 y >20 y	Speech and motor development delay, Rett-like hand movements, loss of developmental gains
*CLN2* *TPP1*	CLN2 disease	tripeptidyl peptidase 1; lysosome	155	c.509-1G > C/p.(Val170Glyfs*29); c.622C > T/p.(Arg208*)	c.851G > T/p.(Glu284Val) in Canada	Congenital/Infantile **Late infantile** Juvenile Late juvenile/Protracted Adult *SCAR7*	0–18 mo **2–4 y** 5–10 y >11 y >20 y >4–11 y	Speech delay, seizures, motor decline
*CLN3*	CLN3 disease	CLN3; lysosome membrane	78	c.461-280_677 + 382del (1-kb deletion)	1 kb intragenic deletion in many countries; 2.8 kb intragenic deletion in Finland	Infantile **Juvenile** Protracted *Autophagic vacuolar myopathy* *Retinitis pigmentosa* *Non-syndromic retinal disease* *Adult cone-rod dystrophy*	0.4 y **5–10 y** >13->40 y >20 y	Rapidly progressing loss of vision, cognitive decline
*CLN4* *DNAJC5*	CLN4 disease	DnaJ homologue subfamily C member 5/CSPα, Cysteine string protein; cytoplasm	3		c.346_348delCTC/p.(Leu116del); c.344T > G/p.(Leu115Arg)	**Adult (autosomal dominant)**	**>20 y**	Seizures, ataxia, behavioural changes
*CLN5*	CLN5 disease	CLN5; lysosome	37		c.1175_1176del/p.(Tyr392*); c.225G > A/p.(Trp75*) in Finland	Congenital Infantile **Late infantile** Juvenile Protracted Teenage Adult	1.5 y **2–4 y** 5–10 y 17 y >50 y	Slowing of psychomotor development, visual failure
*CLN6*	CLN6 disease	CLN6; endoplasmic reticulum membrane	82	c.214G > T/p.(Glu72*)	c.461_463del/p.(Ile154del) in Portugal	**Late infantile** Protracted Teenage Adult Kufs type A *Juvenile cerebellar ataxia* *Progressive myoclonus epilepsy*	**2–4 y** >16 y >20->51 y 7–9 y >15	Speech and motor development delay, seizures
*CLN7* *MFSD8*	CLN7 disease	major facilitator superfamily domain 8-containing protein; lysosome membrane	46	c.881C > A/p.(Thr294Lys)	c.881C > A/p.(Thr294Lys) in Roma Gipsies; c.754+2T>A in Eastern Europe	**Late infantile** Juvenile/late juvenile *Nonsyndromic retinal disease* *Adult macular dystrophy* *Adult cone-rod dystrophy*	**2–4 y** 5–11 y >5 y >29–>65 y >27 y	Speech delay, motor difficulties, seizures
*CLN8*	CLN8 disease	CLN8; endoplasmic reticulum membrane	41		c.70C > G/p.(Arg24Gly) in Finland causing EPMR; c.610C > T/p.(Arg204Cys) and c.789G > C/p.(Trp263Cys) in Turkey	**Late infantile** Juvenile *EPMR/Northern epilepsy*	**2–4 y** 6 y 5–10 y	Language and learning difficulties, motor difficulties, seizures
*CLN10* *CTSD*	CLN10 disease	cathepsin D; lysosome	10			**Congenital** Infantile Juvenile, late juvenile Teenage	**0 y** 0.5–1.5 y 5–11 y 14–15 y	Seizures, spasticity, central sleep apnoea
*CLN11* *GRN*	CLN11 disease	Progranulin, cleaved into granulins; lysosome	3			**Teenage, Adult** *Frontotemporal lobar dementia (when heterozygous)*	**>20 y**	Rapidly progressive visual failure, seizures
*CLN13* *CTSF*	CLN13 disease	cathepsin F; lysosome	11			**Adult Kufs type B**	**>20 y**	Tremor, ataxia, seizures

*Phenotype bold = phenotype caused by complete loss of gene function*.

*Age of onset uses the same age range for each gene: congenital (0–0.5y), infantile (0.5–1.5y), late infantile (2–4 yr), juvenile (5–10 yr), late juvenile (11–12y), teenage (13–19y), adult (20+y)*.

*italics–non-NCL disease phenotype that in some cases may be more typically associated with this gene*.

*CLN9 is not identified. Variations in further genes have been linked with NCL-like phenotypes, these are not included in this table: CLN12/ATP13A2, mutations usually cause Kufor-Rakeb syndrome; CLN14/KCTD7 in cases with infantile and late infantile onset, all other known mutations cause a progressive myoclonic epilepsy or opsoclonus-myoclonus ataxia-like syndrome; SGSH in a case with adult onset, all other known mutations cause MPSIIIA; CLCN6, perhaps modifying disease phenotype (1)*.

*SCAR7, Spinocerebellar Ataxia, Autosomal Recessive 7; EPMR, Epilepsy, Progressive, With Mental Retardation*.

This article summarises the genetic basis of NCL and discusses correlations with disease phenotype. All mutation details can be found in the freely accessible NCL mutation database (www.ucl.ac.uk/ncl-disease).

## Historical Perspective

The concept of NCL as a group of inherited diseases first emerged in the 1960s (2), leading to classification into four broad ages of onset: infantile, late infantile, juvenile, and adult. At this time it was assumed that each of these types was caused by mutations in a different gene, named in advance as *CLN1, CLN2, CLN3*, and *CLN4*, respectively. The first genes to be identified, in the 1990s (*CLN1, CLN2, CLN3*), were responsible for the most common paediatric types. However, these first identified genes were not responsible for all childhood onset cases. For example, the genes *CLN5, CLN6, CLN7*, and *CLN8* cause disease with onset in late infancy like *CLN2*, the first gene identified causing onset at that age (Table 1). *CLN4* was not identified until 2011 (3).

A variety of experimental approaches reflecting the available technology were used to identify NCL genes. The first genes were identified using classic and time-consuming genetic linkage approaches requiring large numbers of similarly affected families followed by positional cloning of the genes [*CLN1* (4) and *CLN3* (5)]. A biochemical approach that detected a missing mannose-6-phosphate tagged lysosomal enzyme in a patient facilitated the identification of *CLN2* (6), alongside ongoing genetic linkage studies. Availability of the human genome sequence meant that going forward fewer families were required to provide sufficient power for genetic linkage analysis, facilitating identification of *CLN5* (7), *CLN6* (8)*, CLN7* (9), and *CLN8* (10). Some genes were identified by recognition of stretches of homozygosity in consanguineous families that narrowed the interval that contained the candidate gene. A gene first identified in an animal model led to identification of *CLN10* (11, 12) in human disease. Improvements in sequencing technology later allowed fast and massively parallel sequencing of the whole exome in single families, and facilitated identification of the remaining disease genes [*CLN4* (3), *CLN11* (13)*, CLN12* (14)*, CLN13* (15)*, CLN14* (16)]. The few families suspected of carrying the putative *CLN9* gene were later found to carry mutations in previously identified NCL genes (17, 18).

As monogenic disorders, each NCL is in effect a separate disease entity. All identified NCL genes lie on autosomes. Most cause disease though classic recessive inheritance, where deleterious mutations are present in disease gene alleles inherited from asymptomatic parents. However, adult onset CLN4 disease is dominantly inherited in the few families described with this disease (1, 3). There are three published reports of uniparental disomy in the NCLs, one in which a patient has complete isodisomy of chromosome 8, leading to homozygosity of a maternally-inherited deletion in *CLN8* (19), and two patients for CLN1, both with paternal isodisomy of chromosome 1 (19, 20).

The majority of NCL genes encode proteins that reside in the endo/lysosomal pathways (1, 21–23). Most are lysosomal proteins—enzymes and soluble proteins (*CLN1/PPT1, CLN2/TPP1, CLN5, CLN10/CTSD, CLN13/CTSF, CLN11*/*GRN*,) or membrane proteins (*CLN3, CLN7/MFSD8, CLN12/ATP13A2*). Two encode endoplasmic reticulum membrane proteins *(CLN6, CLN8*). Other NCL proteins are cytoplasmic (*CLN4/DNAJC5, CLN14/KCTD7*) that peripherally associate with cellular membranes. The *in vivo* substrates for the lysosomal enzymes are incompletely defined, and much remains to be discovered around the functions of the membrane proteins. Nevertheless, recognition of the genetic basis of the NCLs enables the development of targeted therapies even though the underlying disease mechanism for each NCL is not yet fully delineated. It is unlikely that further NCL genes will be identified unless they cause disease in countries where little genetic analysis has been undertaken.

## Genotype-Phenotype Observations

### NCL Classification

A gene-based classification system was codeveloped by international experts in the NCLs (24) that takes into account the full phenotypic consequences that have emerged over the years, and which includes secondary reference to the age of onset. This replaces the former age-based classification in use since the 1960s. It better supports ongoing gene-based therapeutic development.

There is a classic disease phenotype associated with complete loss of function for most NCL genes, with a typical age of onset and disease progression. The age at which first symptoms appear can be used to guide toward which gene(s) may be mutated. For example, clinically similar NCL disease arising from mutations in more than one gene (e.g., what was originally known as variant late infantile onset NCL) can be caused by loss-of-function mutations in *CLN5, CLN6, CLN7*, or *CLN8*.

### Broad Phenotypes

Most NCL genes actually have a wide age of onset and varied disease courses determined by the underlying mutations (Table 1). The increasing implementation of next generation sequencing panels and exome sequencing in diagnosis is leading to more diagnoses of patients with atypical NCL and recognition of these broader phenotypes. These arise from mutations thought or known to have “milder” effects on NCL protein function; and these phenotypes can vary quite considerably. For example, classic CLN6 disease begins in early childhood (late infancy) (8, 25), but disease onset can be delayed as late as adulthood, which also has no associated visual failure (26, 27). Conversely, disease that presents in adulthood caused by mutations in CLN3 may have visual failure as its only or main sign, consistent with this being the presenting symptom for classic juvenile CLN3 disease. Mutations in *CLN7* have been identified in cases of non-syndromic eye disease (28).

This broadening of phenotypes means that disease with a certain age of onset may be caused by loss of function of an NCL protein as well as milder mutations in a gene more usually associated with a younger age of onset. For example, disease beginning in the juvenile age range may be classic CLN3 disease or be juvenile CLN1 disease, or juvenile CLN2, CLN5, CLN6, CLN7, or CLN8 disease.

### Distinct Mutation-Specific Phenotypes

Some mutations cause distinct and varied disease that differs from the phenotypes arising from other mutations in the same gene. For example, a single recessive missense mutation in *CLN8* [p.(Arg24Gly)] (10) causes the phenotype described as progressive epilepsy with mental retardation (EPMR) or Northern epilepsy that is found predominantly in Finland. This disease is very different to typical NCL as it is an intellectual developmental disorder that presents with seizures in the juvenile age range that cease in adulthood, and life expectancy is into late adulthood. It was the first genetic disease to be recognised for *CLN8*, with mutations that cause a more typical NCL described later. Similarly, a missense mutation in *CLN2*/*TPP1* [p.(Val466Gly)] causes a phenotype first described as spinocerebellar ataxia SCAR7. This is a slowly progressing but not life-limiting disease with no ophthalmologic abnormalities or epilepsy, and without typical ceroid/lipofuscin storage (29). A single gain of function missense mutation in CLCN6 has recently been shown to cause very severe disease in children (30) that would not be classed as NCL, although the mouse model lacking the function of the homologous gene causes mild lysosomal storage disease and the CLCN6 gene was considered a candidate gene for mild NCL disease (31).

There is evidence that the most common and very widespread mutation in *CLN3*, a 1-kb deletion found worldwide accounting for ~ 90 percent of the affected alleles in CLN3 disease patients (32) does not completely abolish CLN3 function, indeed it may case a gain of function and therefore disease (33, 34). Due to this deletion dominating reports of CLN3 disease, this led to the suspicion that disease caused by complete loss of CLN3 function may not have been described in humans (33). Other distinct phenotypes have been associated with *CLN3* mutations—these include retinitis pigmentosa without other clinical symptoms, even in mid-late adulthood (35) and a distinct disease described as autophagic myopathy associated with heart failure (36). As predicted (33) the phenotype of *CLN3*-associated disease maybe considerably broader (1). There are reports of other families with mutations in some NCL genes that also have predominantly visual problems (28).

### Overlap With Other Syndromes

Some mutations in NCL genes cause disease that overlaps with other recognised disease syndromes. This has been described for other rare diseases and more common neurological disorders, such as Niemann-Pick C disease with Alzheimer's disease (37), and type 1 Gaucher disease with Parkinson's disease (38).

Mutations in GRN cause diseases with different types of inheritance. A homozygous recessive (bi-allelic) mutation associated with rectilinear profiles, leads to CLN11 disease, whereas mutations present on one chromosome only cause frontotemporal lobar degeneration with TDP-43 inclusions (FTLD-TDP) (13), which is the second most common type of early-onset dementia. The age of onset and neuropathology of FTLD-TDP and NCL are markedly different, yet there are some shared characteristics: there is autofluorescent, NCL-like storage material in the retina, postmortem brain and lymphoblasts of FTLD-TDP patients (39) and in induced pluripotent stem cells from FTLD-TDP patients (40). Progranulin-deficient mice (13) have features of both NCL and FTLP-TDP diseases (41–43). Therefore, autosomal dominant *GRN* mutations in FTLD-TDP patients cause disease through haploinsufficiency, and it is likely that there are shared disease mechanisms underlying disease in adult CLN11 and FTLD-TDP patients.

Some genes identified as causing NCL more commonly cause inherited diseases given different diagnoses. Mutations in *CLN14/KCTD7* cause three different diseases (16, 44–46) classed as progressive myoclonic epilepsy (PME) (47, 48), and in rarer cases PME accompanied by vision loss and lysosomal storage and termed an NCL (16, 49). Mutations in *ATP13A2* typically cause Kufor-Rakeb syndrome and also a late-onset autosomal recessive spastic paraplegia 78 (SPG78) and juvenile onset amyotrophic lateral sclerosis (ALS) (50–52), whereas one family was diagnosed with CLN12 disease (14, 53, 54). Fibroblasts from some SPG78 patients have lysosomal pathology (50). *Atp13a2* knockout mice are reported to accumulate both NCL-type storage material and α-synuclein, and late-onset impairment in sensorimotor functioning. ATP13A2-related disease may therefore represent a disorder with features overlapping both NCL and Parkinson's disease (55). Mutations in *SGSH* usually underlie late infantile onset disease mucopolysaccharidosis type IIIA (MPSIIIA) (56), whereas a mutation in *SGSH* was described in a single case diagnosed with adult onset NCL. Thus, distinctions between inherited disease phenotypes may not be as clear cut as originally anticipated.

There are examples of disease including features of NCL. For example, *CLCN7* underlies a severe autosomal recessive disease combining osteopetrosis, neurodegeneration and lysosomal storage disease (57–59).

### Autosomal Dominant Inherited NCL

The clear recessive nature of most NCL had always suggested that mutation carriers are healthy. Given that disease arises in those who are carriers or carrying compound heterozygous mutations in CLN11/GRN, it may be that carriers of mutations in other NCL genes also have deficits. If so, these are likely to be extremely mild or be very late onset and overlap with common features or ageing, and so have not been linked, even anecdotally.

CLN4 disease (Parry disease) is considered autosomal dominant, with disease manifesting in those carrying one of the three mutations in *CLN4* so far described. Disease in humans caused by complete loss of CLN4 function is not known, although the severity of phenotype in animal models with no CLN4 function (60) would predict those carrying biallelic loss-of-function mutations would have very severe and early onset disease. Disease arising from mutations in CLN4/DNAJC5 may therefore be inherited recessively or dominantly.

### Multi-Gene Disease

There are a few reports of patients carrying changes in more than one NCL gene. One that was later found to be compound heterozygous for mutations in *CLN5* also carries a single mutation in the *CLCN6* gene that causes recessive NCL in animals (31). Another family is reported in which a single mutation in *CLCN6* is the only described variation; a second heterozygous mutation may be present but not identified. In these two families the *CLCN6* carrier parents were healthy. Some patients carry mutations in more than one gene that underlie variant late infantile NCL (47) (i.e., the mutation database lists changes in CLN5 that have been found alongside those in CLN6 or CLN7 or CLN8). These may be examples of a mutation or specific allele of one gene enhancing or ameliorating the NCL disease phenotype. In mouse NCL models, deletion of both cathepsin B and cathepsin L causes disease, but deletion of either gene alone does not (61).

A patient with disease that presented shortly after birth was found to carry heterozygous mutations in CLN5, together with a mutation in POLG1 that acts to maintain mitochondrial DNA integrity (62). Increased expression of CLN8 may act as a modifier of Gaucher disease (63). There may be connexions between the function of NCL genes; for example, GRN interacts with CTSD (40), CLN3 affects trafficking of enzymes to the lysosome (64); CLN5 interacts with CLN2 and CLN3 (65).

Human cancer cells acquire somatic mutations in the NCL genes which may confer a growth advantage (*CLN1/PPT1, CLN2/TPP1, CLN3, CLN4/DNAJC5, CLN5, CLN6, CLN7/MFSD8, CLN8, CLN10/CTSD, CLN11/GRN, CLN12/ATP13A2, CLN13/CTSF, CLN14/*KCTD7, as well as *SGSH*, and *CLCN6*) (www.sanger.ac.uk/genetics/CGP/cosmic/) (66). As more sequence variations are deposited through large-scale genome sequencing projects) (e.g., Exome Aggregation Consortium (ExAC): exac.broadinstitute.org/), further correlations may be revealed.

## Incidence and Prevalence

NCL are considered the most common inherited neurodegenerative disorder of childhood. They occur worldwide, with some forms first recognised in certain geographical regions. Some types are enriched in or absent from certain regions due to historical population (genetic) bottlenecks.

Incidence and prevalence rates are not available world-wide. Incidence rates are probably more robust than estimated prevalence rates, and generally reported between 1 in 14,000 (Iceland) up to 1 in 100,000 (67). The most common NCL in Northern Europe and the UK are juvenile CLN3 disease and late infantile CLN2 disease, but all types are present.

## Diagnostic Implications

### Laboratory Diagnosis

There is an urgency in making an NCL diagnosis now that disease modifying treatments are available or in the pipeline. Biomarkers that follow disease progression and allow the effectiveness of therapies to be monitored are likely to emerge in the near future (68, 69).

New comprehensive approaches are changing the order of diagnostic tests and removing the need for former investigations. Protocols for enzymatic and genetic testing are widely available, making rapid genetic and biochemical diagnosis of most forms of NCL increasingly straightforward (Table 2).

**Table 2:** Summary of NCL gene mutations, patients and families currently contained within the NCL Mutation Database.

**Gene**	**CLN1**	**CLN2**	**CLN3**	**CLN4**	**CLN5**	**CLN6**	**CLN7**	**CLN8**	**CLN10**	**CLN11**	**CLN12**	**CLN13**	**CLN14**	**Grand total**
Mutations	71	155	78	3	37	82	46	41	10	3	1	11	1 (13)	537
No. of patients	230	460	442	12	103	145	109	87	18	4	4	15	3 (21)	1,625
No. of families	177	358	418	*7*	84	118	93	77	13	3	1	9	2 (15)	2,162

*CLN12/ATP13A2: mutations usually cause Kufor-Rakeb syndrome, only those causing NCL are shown here; CLN14/KCTD7: mutations cause NCL with infantile and late infantile onset, all other known mutations cause a progressive myoclonic epilepsy or opsoclonus-myoclonus ataxia-like syndrome. Numbers in brackets are mutations that cause a non-NCL phenotype; they are not included in the overall totals*.

Enzyme testing can rapidly confirm deficiencies of CTSD, PPT1, and TPP1 using saliva, blood samples and dried blood spots (70). These enzyme assays should always be applied in cases with an unusual presentation or later onset, and all diagnoses should be supported by DNA sequencing and mutation analysis where possible. For classic juvenile CLN3 disease, the vacuolated lymphocytes which are a common feature, can be visualised by blood film examination (71).

New DNA technologies now allow testing for many genes in a single step regardless of the presentation (70). NCL genes are part of panels designed to interrogate genes underlying a larger group of syndromic and non-syndromic inherited epilepsies. Some common mutations may be screened by DNA-based testing. This can speed earlier diagnosis of NCL before the appearance of other symptoms and also provides a genetic diagnosis for clinically milder or variant phenotypes. As DNA sequencing leads to the description of multiple genetic variation, the genetic cause of atypical disease for some cases will become clearer. Some patients that previously may have been given a diagnosis of NCL may be demonstrated to have atypical forms of other diseases, and *vice versa*. Carrier detection is not possible by histology and is unreliable by enzyme assay; it should always be based on mutation analysis.

Ultrastructural examination of a skin biopsy or blood sample may be helpful for confirmation of NCL disease for atypical forms that are not enzyme deficiencies or do not receive a genetic diagnosis (Table 1). Extracerebral storage is readily detected in childhood NCLs but not necessarily in NCL presenting in adulthood (27).

### Prenatal Diagnosis

Prenatal diagnosis can be offered to families with a prior history of NCL disease. Preimplantation genetic diagnosis (72) or a combination of enzyme assay and mutational analysis, perhaps with ultrastructural examination of chorionic villus samples obtained at 12–15 weeks gestation, can provide a rapid diagnosis (70).

## NCL in Other Species

Some NCL genes are conserved in unicellular or simple organisms, indicating their fundamental function within eukaryotic cells (73). For example, yeasts contain homologous genes to *CLN1/PPT1, CLN3 CLN10*/*CTSD, CLN12*/*ATP13A2*. The slime mould *Dictyostelium discoideum* particularly expresses further NCL gene homologues or members of gene families (e.g., *CLN2*/*TPP1, CLN4*/*DNAJC5, CLN5, CLN6, CLN7*/*MFSD8* family). NCL also occurs in animals (e.g., dogs, sheep, cows, monkey). Cell and animal models carrying mutations in genes equivalent to those causing human NCL are well used in research. These range from yeasts, up to rodents and other mammals (for clinical development). Some of these models are naturally occurring (e.g., mouse, dog, sheep), others are engineered models (e.g., mouse, pig). Some animal NCL disease is caused by mutations in genes not reported to cause similar disease in humans [*ARSG* in dogs (74), *CLCN6* engineered in mice (31), *CTSB*/*CTSL* engineered in mice as double gene mutations (61)].

## NCL Mutation Database

The NCL Mutation Database (www.ucl.ac.uk/ncl-disease) lists known disease-causing mutations and sequence variations in NCL genes by gene and by individual. Five hundred and thirty-seventh NCL disease-causing mutations are currently listed (Table 2) across >1,625 patients and >2,160 families. Where possible the age of onset, ethnic background and current location, are listed for each family. Data are gathered from case reports or larger collections in clinical or scientific publications, or referred directly, and updated periodically. These vary in detail according to the report source, e.g., case reports usually have more specifics than reports of large group genetic screens. Mutations are mostly described in single individuals or occasionally siblings from the same family. Some mutations are more common in certain populations due to local founder effects. Several NCL genes have widespread distribution across several continents due to ancient founder effects (Table 1).

An estimate of the proportion of cases caused by each mutation can be made, although there is a considerable under-representation of the occurrence of common mutations since the emphasis is on the collation of novel and rare mutations. The most prevalent mutations are the 1 kb deletion in *CLN3* and two mutations in *CLN2* (1).

Correlations can be drawn between genotype, phenotype and morphological changes in patients, and have been reviewed previously (47, 75), for example for CLN2 disease (76). These derived correlations can be used to predict the disease course in a newly diagnosed family.

This database is important (1). The severity of mutations has implications for treatment. It may be important to know if residual protein or function remains. Treatments may be developed that do not fully compensate for complete loss of gene function and can reduce but not completely eliminate the disease burden—these may be sufficient to improve health in families carrying so-called mild mutations but not in individuals lacking all gene function (2). The location of mutations in the protein may highlight key residues and functional or regulatory domains, aiding understanding of protein function (3). The data reveals the relative frequency of mutations; as ultra-rare, found only within certain ethnic groups, or widespread (4). The data is freely available and contained in excel tables that can be downloaded and used by researchers. For example, there is increasing information on frequency of mutations or disease in specific ethnic groups (4). Efficacy of a new treatment may be demonstrated earlier or more robustly if the mutations and their effects on disease progression of the participants are understood. Going forward, functional data for each mutation can begin to be incorporated, as available.

Other databases exist through international cooperation, enabling collection of natural history data for all NCL types and genotype-phenotype data through databases DEM-CHILD (*www.dem-child.eu*) (77, 78). There are disease rating scales (79–81) to follow disease progression. This is increasing understanding of the genetic spectrum of NCL disease as well as provide necessary control data for use in future clinical trials (77).

## Conclusion

Most genes that cause NCL disease in humans are probably identified. This, combined with the broader range of associated phenotypes now described, has shown that the genetic picture is considerably more complex than was first envisioned at the start of the genetic era of the NCL. The functions of all NCL genes and thereby disease mechanisms are not yet known. As understanding increases overlap with other rare and common diseases, such as retinal dystrophies may indicate shared disease mechanisms (82).

The gene dosage or the specific mutations show correlation with clinical phenotype. Some variation in clinical phenotype is therefore explained by differing levels of residual protein function. However, variation between families and even siblings shows that co-inheritance of other genetic variations could influence disease phenotype. It is still unclear whether the underlying pathogenic mechanisms are partly shared between classic NCL forms and the alternative disease forms.

The era of genomic medicine is approaching, where genomic information will be used to design the best clinical care for an individual. For the NCL, personalised treatment approaches will be tailored to the underlying mutation and the genetic background of each patient. An early example is the design and delivery of an oligonucleotide therapy for a child with CLN7 disease (83).

Therapeutic development beyond current palliative treatments is advancing slowly. This relies on continued collection of natural history data for the broadening NCL spectrum to provide a control cohort to aid design of future clinical trials. The first approved treatment is for children with classic late infantile CLN2 disease which delivers recombinant protein directly into the brain at regular intervals. For the best long-term clinical benefit for any NCL disease, treatment must begin as early as possible, before any symptoms, which requires rapid and earlier diagnosis using genotype. This may be facilitated by advances in DNA-based approaches that allow future newborn screening (84, 85).

## Author Contributions

SM devised, interpreted the data, and wrote the review. EG collated the data on mutations, genes and phenotype, and contributed to the writing. All authors contributed to the article and approved the submitted version.

## Funding

This work was funded by the Medical Research Council (MRC), award MR/V033956. SM (ORCID:0000-0003-4385-4957) reports support by Biomarin for maintaining the NCL mutation database. The support of UCL and the MRC that provides funding to the MRC Laboratory for Molecular Cell Biology University Unit at UCL (award code MC_U12266B) toward lab and office space is acknowledged. All research at Great Ormond Street Hospital NHS Foundation Trust and UCL Great Ormond Street Institute of Child Health is made possible by the NIHR Great Ormond Street Hospital Biomedical Research Centre. The views expressed are those of the authors and not necessarily those of the NHS, the NIHR or the Department of Health.

## Conflict of Interest

The authors declare that the research was conducted in the absence of any commercial or financial relationships that could be construed as a potential conflict of interest.

## Publisher's Note

All claims expressed in this article are solely those of the authors and do not necessarily represent those of their affiliated organizations, or those of the publisher, the editors and the reviewers. Any product that may be evaluated in this article, or claim that may be made by its manufacturer, is not guaranteed or endorsed by the publisher.
